# Cardiovascular Risk and Lifestyle: comparison between teaching workers in Portugal and Brazil

**DOI:** 10.1590/0034-7167-2023-0354

**Published:** 2024-06-14

**Authors:** Noeli das Neves Toledo, Gilsirene Scantelbury de Almeida, Nair Chase da Silva, Luana Coimbra, Sara Alves Monteiro, Anna Camily Oliveira Bitar, Filipa de Brito Homem, Irma Brito

**Affiliations:** I Universidade Federal do Amazonas. Manaus, Amazonas, Brazil; II Centro Hospitalar e Universitário de Coimbra EPE, Unidade de Cuidados Intensivos Cardíacos. Coimbra, Portugal; III Escola Superior de Enfermagem de Coimbra & UICISA. Coimbra, Portugal

**Keywords:** Heart Disease Risk Factors, Life Style, Universities, Occupational Health, Community-Based Participatory Research, Factores de Riesgo de Enfermedad Cardiaca, Estilo de Vida, Universidades, Salud Laboral, Investigación Participativa Basada en la Comunidad

## Abstract

**Objective:**

Compare Cardiovascular Risk between workers in Brazil and Portugal who work in the teaching context and its relationship with Lifestyle and Common Mental Disorder.

**Methods:**

Cross-sectional study that compared the cardiovascular health conditions of teaching workers in Manaus (Brazil) and Coimbra (Portugal). The odds ratio between groups was estimated.

**Results:**

The differences were: Smoking and hypercholesterolemia in participants from Portugal. Hypertension, chronic disease, increased abdominal perimeter, common mental disorder, and absence from work in Brazil. The variables with the greatest effect for high cardiovascular risk were: Country-Portugal [17.273 (95%CI1.538-193.951)], sex-male [61.577 (95%CI5.398-702.469)] and smoking [593.398 (95%CI57.330-6.142.020)].

**Conclusion:**

The differences in risk between groups showed that participants from Portugal, men, with high blood pressure and/or smokers are the most vulnerable to having a cardiovascular event. There is a need for interventions to promote cardiovascular health in the workplace in both countries.

## RESUMEN

**Objetivo:**

Comparar el Riesgo Cardiovascular entre trabajadores de Brasil y Portugal que actúan em el contexto docente y su relación con el Estilo de Vida y el Trastorno Mental Común.

**Métodos:**

Estudio transversal que comparo las condiciones de salud cardiovascular de trabajadores docentes de Manaos (Brasil) y Coimbra (Portugal). Se estimó el odds ratio entre grupos.

**Resultados:**

Las diferencias fueron: Tabaquismo e hipercolesterolemia en participantes de Portugal. Hipertensión, enfermedad crónica, aumento del perímetro abdominal, trastorno mental común y ausentismo laboral en Brasil. Las variables com mayor efecto para alto riesgo cardiovascular fueron: País-Portugal [17.273 (IC95%1.538-193.951)], sexo-masculino [61.577 (IC95%5.398-702.469)] y tabaquismo [593.398(IC_95%_57.330- 6.142.020)].

**Conclusión:**

Las diferencias de riesgo entre grupos mostraron que los participantes portugueses, los hombres, los hipertensos y/o fumadores son los más vulnerables a sufrir un evento cardiovascular. Es necesario realizar intervenciones para promover la salud cardiovascular en el lugar de trabajo en ambos países.

## INTRODUCTION

Data reveal that Cardiovascular Diseases (CVD) are among the main causes of mortality, accounting for 31% of all causes of death in the world. According to WHO estimates, 85% of deaths were caused by acute myocardial infarction (AMI) and stroke. Although the prevalence is higher in low- and middle-income countries, it is noteworthy that, among the 17 million deaths from chronic non-communicable diseases in the world, 37% belong to the group of CVDs and affect people under 70 years old^(1,2)^.

Although gender, age, heredity and some chronic diseases (Hypertension, Diabetes, Dyslipidemia and Obesity) are considered cardiovascular risk factors (CVRF), socioeconomic conditions (income, education level, profession) and lifestyle (use tobacco, alcohol, inadequate diet and sedentary lifestyle) are potentially more relevant to be faced when the focus is on promoting the health and well-being of the social group^(3)^. This perspective is aligned with the concept of modifiable risk factors.

In Portugal and Brazil, estimates showed that the population has a high prevalence of contextual CVR factors, mainly with regard to the adoption of inappropriate habits and poor control of blood pressure, blood glucose and cholesterol values^(3,4)^. As cardiovascular events have a higher incidence from middle age onwards, that is, in full active and productive age. This problem falls within the scope of worker health. However, despite there being occupational health programs with mandatory exams, aspects of health and well-being are little explored or neglected, especially with regard to the assessment of CVR and indicators of physical or mental well-being.

Although Portugal and Brazil have their own sociocultural identities, health care needs are considered to be similar, especially with regard to the protection of CVR factors^(3,5,6)^.

In relation to workers’ health, aspects of health and well-being are little known or are being neglected, especially with regard to the prevention of physically and mentally disabling diseases. There is evidence that this scenario has worsened due to the COVID-19 pandemic^(7,8)^. Studies carried out in different locations around the world showed that the population suffered from illness and complications resulting from coronavirus contagion, as well as changes in their daily social life, especially with regard to the way work processes are organized and access to health services^(9,10,11,12)^. School workers are among the professional categories most impacted by such transformations, especially when considering that they experienced an accelerated process of adaptation both in working remotely, during the confinement period, and in the resumption of teaching/learning activities so that, thus, schools could function satisfactorily from a quantitative and qualitative point of view^(13)^. It is important to highlight that the need to restructure the teaching-learning process, forms of psycho-pedagogical and social care – demanded by students who also suffered from the new living conditions imposed by the pandemic – compromised the health and well-being of these workers in recent post-pandemic years. From this perspective, this study aims to better understand the cardiovascular health conditions of school workers, working in two different locations: Manaus, in the state of Amazonas (Brazil) and Coimbra (Portugal), taking into account that in both countries there is no evidence from studies on associated risk factors among school workers.

When considering that salutogenic contexts are spaces that generate more health and prevent illness, this study aligns with the proposal for Participatory Action Research in Health embodied in the PEER--IESS model (Participation, Engagement, Empowerment and Research for Innovation and Expansion of Salutogenic Settings) of salutogenic Higher Education Institutions^(14,15)^. Portugal and Brazil established a multicenter study partnership in a school context. The partnership aims to activate dialogic and creative strategies to mobilize the target audience^(14,16,17)^, for research actions that allow for the situational diagnosis of workers’ lifestyle and well-being and the co-creation of salutogenic strategies. In this context, it was agreed to carry out a cross-sectional study based on Cardiovascular Risk (CVR) screening, verifying possible relationships with Lifestyle and the presence of Common Mental Disorder.

## OBJECTIVE

Compare Cardiovascular Risk between workers in Brazil and Portugal who work in the teaching context and its relationship with Lifestyle and Common Mental Disorder.

## METHODS

### Ethical aspects

The study was conducted in accordance with national and international ethics guidelines, and was approved by the Ethics Committees of the Federal University of Amazonas (UFAM), Brazil, and the Escola Superior de Enfermagem de Coimbra (ESEnfC), Portugal. Free and Informed Consent was obtained from all individuals involved in the study online.

### Study design, period and setting

This is an epidemiological, observational study that used the recommendations of the Strengthening the Reporting of Observational Studies in Epidemiology (STROBE) checklist, adapted to the type of study^(18)^.

It is part of a multicenter project PEER-IESS (Participation, Engagement, Empowerment and Research for Innovation and Expansion of Salutogenic Settings), involving Educational Institutions in Brazil and Portugal^(14,15,19,20)^. The results of a Cardiovascular Risk screening and its relationship with Lifestyle and Common Mental Disorder among workers who worked in the teaching context are described. Data collection was carried out between August 2022 and May 2023 in two different locations (Manaus – Brazil, and Coimbra – Portugal).

### Population, inclusion and exclusion criteria

The study was carried out with workers who worked in the teaching context (teaching and non-teaching staff), in two different countries (Brazil and Portugal, as already mentioned), which were adopted as the universe of the target population that met the inclusion criteria and exclusion. Therefore, all workers from the respective countries and institutions involved were invited to carry out an assessment of their health condition. Pregnant women and other workers who were on leave for any reason (vacation, maternity/paternity leave, training or illness) were excluded. The calculation of the estimated sample considers the anticipated frequency of 50%, the confidence limit of 5%. The design effect for studies with random samples of 1.0 was 297 for workers in Brazil and 197 for Portugal.

In order to involve the target audience in screening activities, invitations were sent to the workers’ institutional email, at least twice a week, during the data collection period (September/2022 to June/2023). In parallel, other mobilization strategies were also adopted (short video, webinar, digital leaflet and posters). The resources were made available on the websites and internal communication networks of each institution involved. Despite all efforts and strategies to reach the sample size, the quantity was not reached.

When considering the universe of workers in each country, the sample was constituted in a random and non-probabilistic way, so that all workers could choose whether or not to participate in the study, totaling 203 participants, 94 from Brazil and 110 from Portugal.

### Study protocol

Data collection was carried out by health professionals and nursing students, all previously trained in a training session.

The first moment of data collection consisted of answering the instrument that contained questions about sociodemographic and work conditions, individual and family health history, as well as lifestyle (LS) and screening for Common Mental Disorder - CMD. To assess lifestyle, the questionnaire called: FANTASTIC Lifestyle was used, consisting of 25 questions, subdivided into nine domains, which are: 1) family and friends; 2) physical activity; 3) nutrition; 4) cigarettes and drugs; 5) alcohol; 6) sleep, seat belts, stress and safe sex; 7) type of behavior; 8) introspection and 9) work. The alternatives are in the form of a Likert scale or dichotomous, with the alternative on the left always being the one with the lowest value, representing less association with a healthy lifestyle. The sum of the points allows us to arrive at a total score that categorizes individuals into five groups: excellent (85 to 100 points), very good (70 to 84 points), good (55 to 69 points), regular (35 to 54 points) and needs improvement (0 to 34 points). The lower the score, the greater the need for lifestyle modification^(21)^. The CMD assessment was carried out using the Self Reporting Questionnaire (SQR-20). In the adapted version, the first 20 items aim to investigate non-psychotic morbidities, such as: fatigue, insomnia, forgetfulness, irritability, difficulty concentrating, somatic complaints, depressive/anxious mood, decrease in vital energy and depressive thoughts. The score can range from 0 (no probability) to 20 (extreme probability) of having CMD, with a cutoff point of 5 for females and 7 for males^(22,23,24,25)^.

The second stage of data collection consisted of evaluating the following biosignals: blood glucose, cholesterol, blood pressure, weight, height and abdominal perimeter.

For capillary measurements of blood glucose and total cholesterol, a portable device was used, with a blood sample obtained from a puncture in the pulp of the participant’s index finger. The puncture device (lancet) was for individual use and disposable. Although the test values do not constitute a medical diagnosis, the following reference parameters were adopted:

Normal blood glucose (>126 mg/dl) for participants who reported fasting ≥8 hours. For those who reported not fasting, blood glucose was considered increased from values >200 mg/ dl^(26)^. Cholesterol was considered increased when the participant presented values ≥ 190 mg/dl, regardless of fasting time. In all conditions with altered values, the participant was instructed to repeat the test^(27)^.

Blood pressure (BP) was measured using a digital arm device. Participants considered to have high blood pressure were those who had systolic blood pressure (SBP) values ≥ 140 mmHg and/ or diastolic blood pressure (DBP) ≥ 90 mmHg during the casual measurement^(28)^.

Participants who reported having a diagnosis of arterial hypertension (AH) were considered in the prevalence calculation, even if the casual measurement of their BP was within normal values.

Height was measured using a portable stadiometer and weight was measured using a portable scale, with a maximum capacity of 150kg. Participants were instructed to step onto the scale barefoot, and the weight of their clothes (1 kilo) was estimated to reduce their body weight. BMI was classified as normal (values between 18.5 and 24.9 kg/m2) or increased (values ≥ 25 kg/m2)^(29)^.

Waist circumference was measured using a non-extensible plastic measuring tape (size 1.5 m). The measurement was taken on the circumference of the trunk, at the navel line. The Metabolic Risk for men was classified as: Low risk (BP < 94 cm), Increased risk (BP ≥ 94 cm). The same classification for Women with slightly lower cutoff points: Low risk (BP < 80 cm), Increased risk (BP≥ 80 cm)^(29)^.

After evaluating biosignals, cardiovascular risk (CVR) was calculated. The European Society of Cardiology recommends that global CVR should be estimated, using the SCORE (Systematic Coronary Risk Evaluation), in apparently healthy people over 40 years of age. SCORE is the probability of fatal cardiovascular events in 10 years and depends on age, gender, hypertension, dyslipidemia and smoking. Portugal is part of the set of countries with an estimated low risk, with the following risk classification being adopted: Low (< 1%); Moderate (≥1% and <5%); High (≥5% and <10%); Very high (≥10%)^(30)^.

Although the standards in Brazil are slightly different, considering the comparison of results between Portugal and Brazil, it was decided to adopt the values of the European Society of Cardiology for all participants, from both countries. Participants received verbal and written information on the values of each of the measurements taken and personalized advice regarding their cardiovascular risk.

### Analysis of results and statistics

From the data collected in Excel tables that, after being categorized, were transferred to SPSS, a simple descriptive statistical analysis was performed, calculating the difference between groups using Pearson’s Chi-square test, considering a significance level of 5% and p-value. ≤ 0.05. The effects of the variables Cardiovascular Risk, Lifestyle and Common Mental Disorder were estimated by odds ratio, obtained by multinomial logistic regression, with bivariate variance estimated by OddsRatio (OR). The collinearity test was also carried out, which was absent for all variables in the model. Odds ratios were presented with a 95% confidence interval and a p value ≤ 0.05. All data was analyzed with the help of a statistical professional

## RESULTS

When comparing sociodemographic conditions between workers in Brazil and Portugal, it is possible to observe differences in terms of the average number of children, the practice of faith and family income. It is worth highlighting the differences in the values of the Real (Brazil) and Euro (Portugal) currencies (R$1.00 to €5.5) and their respective Minimum Wages (SM). Thus, the percentage of workers in Brazil who reported an income ≥ 8 MW (58.1%) is approximately equivalent to the percentage of those in Portugal who reported an income between 1-3 MW (80%) (Table 1).

**Table 1:** Sociodemographic conditions of workers in Portugal and Brazil, according to the variables: sex, marital status, children, practice of faith, family income and income dependents. Portugal – Coimbra and Manaus-Brazil, 2022-2023

Variables	Country		Total 203(100)	p value
	Portugal 110(54.2)	Brasil 93(45.8)		
Gender^n(%)^				0.116
Male	48 (43.6)	32 (34.4)	80 (39.4)	
Female	62 (56.4)	61 (65.6)	123 (60.6)	
Age(Md/Max-Min)	42 (22-65)	44 (24-67)	0.142	
Marital status^n(%)^				0.108
Without a partner	28 (25.5)	32 (34.4)	60 (29.6)	
With a partner	82 (74.5)	61 (65.6)	143 (70.4)	
Children^n(%)^				0.551
Yes	72 (65.5)	61 (65.6)	133 (65.5)	
No	38 (34.5)	32 (34.4)	70 (34.5)	
Number of children^(Md/Max-Min)^	1 (0-4)	2 (0-4)	-	0.019
Religious person^n(%)^				
Yes	55 (50)	70 (24.7)	125 (61.6)	**<0.001**
No	55 (50)	23 (75.3)	78 (38.4)	
Family in come^n(%)^				
1-3 Minimum Wages^1^	88 (80)	9 (9.7)	97 (47.8)	**<0.001**
4-7 Minimum Wages	20 (18.2)	30 (32.3)	50 (24.6)	
≥ 8 Minimum Wages	2 (1.8)	54 (58.1)	56 (27.6)	
Dependentes da Renda (Md/Max-Min)	2 (1-6)	2 (1-6)	-	0.873

1 *The minimum wage per month in Brazil corresponds to R$ 1.412,00 reais or U$ 291,65 American dollars according to the Central Bank of Brazil on December 29th, 2023.*

Regarding working conditions, the majority of participants in Brazil were teachers (54.8%), while, in Portugal, the majority were technical professionals who worked in the teaching context (66.4%). The length of employment at the institution was longer in the Brazilian group [10 (1-43) years]. The need to take time off from work activities was more frequent among participants from Brazil (31.2%), the majority being due to illness (82.8%), as shown in Table 2.

**Table 2:** Work characteristics of participants from Portugal and Brazil, according to job function and length of employment at the institution, as well as means of transport, need and reason for absence from work in the last year. Portugal – Coimbra and Manaus-Brazil, 2022-2023

Variables	Country		Total 203(100)	p value
	Portugal 110(100)	Brazil 93(100)		
Labor Activity^n(%)^				0.002
Teacher	37 (33.6)	51 (54.8)	88 (43.3)	
Not a teacher	73 (66.4)	42 (45.2)	115 (56.7)	
Time at the Institution^(Md/Max-Min)^	7 (1-44)	10 (1-43)	-	0.042
Means of transport^n(%)^				0.481
Car/motorcycle	98 (89.1)	84 (90.3)	182 (89.7)	
Bus or Van	8 (7.3)	8 (8.6)	16 (7.9)	
Walking	4 (3.6)	1 (1.1)	5 (2.5)	
Leave from work^n(%)^				0.01
Yes	18 (16.4)	29 (31.2)	47 (23.2)	
No	92 (83.6)	64 (68.8)	156 (76.8)	
Reason for Leave^n(%)^				0.003
Disease	7 (38.9)	24 (82.8)	31 (66)	
Others	11 (61.1)	5 (17.2)	16 (34)	

Regarding the health profile, it can be seen in Table 3 that data on increased cholesterol, tobacco consumption and moderate and high CVR indicated higher rates in the group from Portugal. While self-reported AH, history of chronic disease and the highest risk of metabolic disease was in the group from Brazil. The group from Brazil was the one that most reported having worsened physical activity and sexual behavior, as well as improvements and worsening in their diet. Furthermore, 83.9% of participants from Brazil did not notice an improvement in any of their habits in the last 2 years. The group from Portugal had higher percentages of habits that improved (physical activity and sleep quality). Although the groups obtained scores on the EVF considered “Very Good” (between 70 and 84 points), the highest average was among workers in Portugal (74.39±9 points). It is noteworthy that almost half of the participants from Brazil (44.1%) were classified as having a Regular or Improving lifestyle. When considering differences by sex, the median CMD assessment points were slightly higher in the Brazilian group. However, the highest prevalence of the presence of CMD was among participants from Brazil (38,7%).

**Table 3:** Health profile of workers in Portugal and Brazil, according to self-assessment of health, habits and measurements (metabolic, blood pressure and anthropometry), as well as lifestyle assessment and screening for mental disorders and Cardiovascular Risk. Portugal – Coimbra e Manaus-Brazil, 2022-2023

Variables	Country		Total 203(100)	p value
	Portugal 110(100)	Brazil 93(100)		
Self-assessment of health condition^n(%)^				0.031
Improving/Average	71 (64.5)	47 (50.5)	118 (58.1)	
Very good/Excellent	39 (35.5)	46 (49.5)	85 (41.9)	
Previous Illness^n(%)^				0.014
Yes	29 (26.4)	39 (41.9)	68 (33.5)	
No	81 (73.6)	54 (58.1)	135 (66.5)	
Types of Illness^n(%)^				0.277
Cardiovascular Disease	10 (34.5)	9 (47.4)	19 (39.6)	
Other Chronic conditions	19 (65.5)	10 (52.6)	29 (60.4)	
Tobacco Consumption^n(%)^				**<0.001**
Yes	31 (28.2)	6 (6.5)	37 (18.2)	
No	79 (71.8)	87 (95.5)	166 (81.8)	
Casual Blood Glucose Measurement^n(%)^				0.563
Normal	108 (98.2)	92 (98.9)	200 (98.5)	
Increased	2 (1.8)	1 (1.1)	3 (1.5)	
Self-Referred Diabetes^n(%)^				0.063
Yes	3 (2.7)	8 (8.6)	192 (94.6)	
No	107 (97.3)	85 (91.4)	11 (5.4)	
Prevalence of Diabetes^n(%)^	5 (4.5)	8 (8.6)	13 (6.4)	0.187
Casual Cholesterol Measurement^n(%)^				0.008
Normal	71 (64.5)	75 (80.6)	146 (71.9)	
Increased	39 (35.5)	18 (19.4)	57 (28.1)	
Casual Blood Pressure Measurement^n(%)^				0.471
Normal	80 (72.7)	69(74.2)	149(73.4)	
Increased	30 (27.3)	24(25.8)	54(26.6)	
Self-reported hypertension^n(%)^				0.005
Yes	7 (6.4)	18 (19.4)	25 (12.3)	
No	103 (93.6)	75 (80.6)	178 (87.7)	
Prevalence of Hypertension^n(%)^	31 (28.2)	35 (37.6)	66 (32.5)	0.1
Risk of Metabolic Disease^n(%)^				0.017
No	74 (67.3)	48 (51.6)	122 (60.1)	
Yes	36 (32.7)	48.4 (45)	81 (39.9)	
Cardiovascular risk^n(%)^				0.002
Low	59 (53.6)	65 (69.9)	124 (61.1)	
Moderate	36 (32.7)	27 (29)	63 (31)	
High/Very High	15 (13.6)	1 (1.1)	16 (7.9)	
Habits that improve^n(%)^				
Food	42 (38.2)	48 (51.6)	90 (44.3)	0.038
Physical activities	46 (41.8)	6 (6.5)	52 (25.6)	**<0.001**
Sleep habits	21 (19.1)	6 (6.5)	27 (13.3)	0.006
Sexual habits	5 (4.5)	6 (6.5)	11 (5.4)	0.385
Alchool/drug consumption	6 (5.5)	12 (12.9)	18 (8.9)	0.053
None	24 (21.8)	78 (83.9)	102 (50.2)	**<0.001**
Habits that got worse^n(%)^				
Food	6 (5.5)	45 (48.4)	51 (25.1)	**<0.001**
Physicalactivities	18 (16.4)	45 (48.4)	63 (31)	**<0.001**
Sleep habits	42 (38.2)	45 (48.4)	87 (42.9)	0.093
Sexual habits	1 (0.9)	6 (6.5)	7 (3.4)	0.037
None	50 (45.5)	45 (48.4)	95 (46.8)	0.391
Lifestyle Score^– FANTASTICM(DV)^	74.39 (9)	69.60 (9.2)	-	**<0.001**
Lifestyle Classification^FANTASTIC n(%)^				0.005
Excellent to good^(>69 points)^	82 (74.5)	52 (55.9)	134 (66)	
Regular to Better^(≤69 points)^	28 (25.5)	41 (44.1)	69 (34)	
Common Mental Disorder Score^Md (Max-Min)^	3 (0-15)	4 (0-17)	-	0.049
Yes n(%)	28 (25.5)	36 (38.7)	139 (68.5)	0.031
No n(%)	82 (74.5)	57 (61.3)	64 (31.5)	

The variables that had a significant simple association with the CVR variable were (Table 4): country [high risk (p < 0.007)], sex [moderate and high risk (p < 0.001)], income [moderate risk (p < 0.017 ) and high (p < 0.021)], income-dependent [high risk (p < 0.010)], BMI [moderate risk (p < 0.027) and high risk (p < 0.042)], metabolic disease risk [moderate risk (p < 0.001) and high (p < 0.014)], SAH [moderate risk (p < 0.001), SBP [moderate and high risk (p < 0.001)], PAD [moderate risk (p < 0.001) and high risk (p < 0.012 )], Tobacco [moderate and high risk (p < 0.001)], Worsened sleep [high risk (p < 0.016)], Fantastic Lifestyle [moderate risk (p < 0.009)] and Cholesterol [moderate risk (p < 0.016) and high(p < 0.001)].

**Table 4:** Crude analysis of factors associated with Cardiovascular Risk (Low, Moderate, High) of workers in Portugal and Brazil. Portugal – Coimbra e Manaus-Brazil, 2022-2023

Variables	^#^OR-^##^RCV Moderate vs Low ^#^OR adjusted ^[IC95%]^	p value	^#^OR ^##^RCV High vs Low ^#^ OR adjusted ^[IC95%]^	p value
Country(Portugal)	1.46 (0.797-2.706)	<0.217	16.525 (2.117-128.972)	<0.007
Gender^(Male)^	9.968 (4.937-20.126)	<0.000	69.545 (20.723-554.459)	<0.000
Income^(SM)^				
1-3	2.560 (1.181-5.5450)	0.017	11.408 (1.433-90.840)	0.021
IncomeDependents	0.768 (0.593-0.996)	0.046	0.488 (0.282-0.844)	0.010
BMI*	1.075 (1.008-1.147)	<0.027	1.107 (1.004-1.221)	<0.042
AP**	1.047 (1.020-1.074)	<0.001	1.051 (1.010-1.093)	<0.014
AH(No)***	0.189 (0.097- 0.368)	<0.000	556 (0.177-1.745)	<0.314
SBP^▴^	1.066 (1.041-1.092)	<0.000	1.067 (1.030-1.105)	<0.000
DBP^▴▴^	1.063 (1.028-1.098)	<0.000	1.067 (1.014-1.122)	<0.012
Tobacco ^(No)^	0.053 (0.015-0.188)	<0.000	0.004 (0.001-0.023)	<0.000
Worst Sleep	6.135 (0.610-0.114)	<0.689	233 (0.071-0.763)	<0.0016
EVF^+^	0.957 (0.925-0.989)	0.009	990 (0.935-1.048)	<0.0724
cholesterol	1.12 (1.002-1.022)	0.016	1.041 (1.025-1.057)	<0.0000

^#^Odds Ratio (OR); ^##^Cardiovascular risk; ^*^Body Mass Index (BMI); ^**^Abdominal Perimeter (AP); ^***^Arterial Hypertension (AH); ^▴^Systolic Blood Pressure (SBP);^▴▴^ Diastolic Blood Pressure (DBP); + FANTASTIC Lifestyle (EVF)

In Table 5 are the results of the final Multivariate Regression model, and it is possible to observe that the factors associated with high and moderate CVR were the same, with substantial differences in the effect values (Odds Ratio – OR). Participants from Portugal presented, respectively, odds ratios for high and moderate cardiovascular risk of 17.273 (95%CI 1.538- 193.951)] and 1.882 (95%CI 0.772-4.589). Male participants had a higher odds ratio, with 61.577 (95%CI 5.398-702.469)] for high risk, and 9.458 (95%CI 3.877-23.077) for moderate risk. Those who reported being smokers had an extremely high odds ratio for high cardiovascular risk 593.398(95%CI 57.330-6.142.020)] and moderate [50.594 (95%CI 9.430-271.462).

**Table 5:** Adjusted analysis, using Multivariate Regression, of factors associated with Cardiovascular Risk (Low, Moderate and High) of workers in Portugal and Brazil. Portugal – Coimbra and Manaus-Brazil, 2022-2023

Variables	^#^OR-^##^RCV Moderate vs Low ^#^OR adjusted ^[IC95%]^	p value	^#^OR ^##^RCV High vs Low ^#^ OR adjusted ^[IC95%]^	p value
Country^(Portugal)^	1.882 (0.772-4.589)	<0.164	17.273 (1.538-193.951)	<0.021
Gender^(Male)^	9.458 (3.877-23.077)	<0.000	61.577 (5.398-702.469)	<0.001
SBP▴	1.086 (1.051-1.122)	<0.000	1.126 (1.060-1.196)	<0.000
Tobacco ^(No)^	50.594 (9.430-271.462)	<0.000	593.398(57.330-6142.020)	<0.000

▴ *Systolic Blood Pressure (SBP).*

## DISCUSSION

When comparing groups, the findings showed that participants from Brazil had higher prevalence of self-reported AH (19.4%), history of chronic disease (41.9%) and increased metabolic risk (45%). In Brazil, the prevalence of CVR factors varies depending on the region of the country, and it is possible to find much higher prevalences of AH (39.3%) and Dyslipidemia (64.25%)^(4,31,32)^. Specifically regarding the prevalence of AH, a telephone survey carried out in the main Brazilian capitals identified that throughout 2017, 1 in 4 adults died as a result of complications caused by the disease^(4)^.

When comparing national data from both countries with other factors that predispose the development of AH and other CVDs, it appears that the frequencies of pre-obesity (57.2%), obesity (22.4%), inadequate diet (18.2%) and DM (9.1%) are proportionally lower in relation to the percentages in Portugal [nutrition considered inadequate (71.3%), pre-obesity/obesity (62.1%)], except DM whose percentage is slightly lower (8.9%)^(3,4)^.

Other important findings from participants in Portugal are the prevalence of increased cholesterol (35.5%) and tobacco consumption (28.2%), which were higher compared to participants in Brazil (19.4%/6. 5%) and with national data from Portugal (31.5% and 25.5% respectively). On the other hand, the prevalence of participants from Portugal who reported having a diagnosis of AH (19.4%) was much lower than national data from Brazil (39.3%) and Portugal (43.1%)^(3)^.

Considering the degradation of lifestyles over the last 2 years in the two groups investigated, we can infer that it may be related to the pandemic period, highlighting the need to implement salutogenic measures, that is, for the work environment to develop personal and collective that lead workers to adopt good self-care practices and, consequently, achieve better health potential. It is urgent to support people to reestablish healthy habits and avoid the development of chronic diseases, such as: obesity, hypertension and DM^(11,12)^.

Regarding the risk of cardiovascular events in the next 10 years^(30)^, we identified that participants from Portugal had higher percentages of moderate (32.7%) and high (13.6%) CVR when compared to those from Brazil. The variables associated with an increase in the odds ratio of high and moderate cardiovascular risk were among male participants from Portugal and, mainly, those who reported consuming tobacco.

An epidemiological study, carried out in Portugal, concluded that the 5 factors of CVR are: pre-obesity/obesity, hypertension, dyslipidemia, low level of physical activity and smoking, with more than half of the population (68%) having at least ≥ 2 factors of CVR, in which AH and DM are considered diseases that are more difficult to control^(3)^. In the Brazilian reality, estimates showed that CVR increased with age and in the low-income population. Hypertension, hypercholesterolemia, diabetes and smoking are factors in CVR in both sexes^(33)^.

The data from this study reveal two important focuses of attention for interventions to promote health and well-being: the lack of adherence to a healthy lifestyle, especially with regard to tobacco use. Participants from both countries presented a vulnerable condition with regard to cardiovascular health. Although risk factors are widely described in the scientific literature, screenings to evaluate CVR are seen as enhancing strategies in the prevention and control of these diseases, as they optimize the implementation of early treatment, especially in people at higher risk^(31,32,33,34,35)^. When considering that tobacco consumption is a problem not only for the group of participants from Portugal, but for the largest portion of the population of both nations, including among the youngest, it is understood that implementing and promoting smoking cessation interventions is one of the strategies that drastically reduce the likelihood of CVD^(36)^.

The study that evaluated cardiovascular (CV) health and its relationship with stress in university workers in Rio Branco - Brazil identified that none of the participants met the criteria for ideal CV health (diet, physical activity, BMI, smoking, hypertension, diabetes and hypercholesterolemia). The majority (91%) were classified as having low CV health and work stress was associated with obesity [OR 2.11 (95% CI 1.06-4.22; p = 0.034)] and inadequate diet: [OR 2.31 (95% CI: 1.29-4.13 p = 0.005)]^(37)^.

Furthermore, another international survey, which compared the CV health of the population of 5 high- and low-income countries (England, USA, Brazil and Ethiopia), identified that Brazil obtained the lowest score in CV health (7.7/12), with only 38.7% of the population considered to have ideal CV health. In contrast, Ethiopia scored highest (10.5/12) as 91.2% of the population was in ideal CV health. When comparing findings from Brazil with high-income countries (England and USA), the ideal targets for BMI (42.5%), total cholesterol (63.7%) and smoking (84.5%) were achieved with greater prevalence in Brazil. On the other hand, the USA surpasses Brazil in terms of the percentage of people with normal BP values (43.5%) and an adequate level of physical activity (59.8%). England had the best performance among the three nations in the variables: blood glucose (75.5%), physical activity (70%) and BP (66.3%)^(38)^.

In this context, it is highlighted that the Health Promoting Universities (UPS) movement, by allowing the strengthening of an organizational culture of care, involving the university community, can promote multiple and recurring actions that contribute to adherence to a better lifestyle. According to the PEER-IESS model^(19,39)^, educational institutions can also add value to the principle of inseparability between teaching, research and extension, when they develop or promote interventions based on the Participatory Action-Research in Health (PaPS) approach. This approach goes beyond the collection of scientific data because it proposes the adoption of dialogical and creative strategies that enhance the university community’s ability to face its problems, increase its health literacy and make environments more salutogenic^(15,^40^)^.

### Study Limitations

The different adherence of workers was considered as a limitation of the study: teachers from Portugal and administrative technicians from Brazil. Although the sample size estimate was not reached, the findings in the sample are worrying, making it possible to generalize that CVR factors are similar among workers who work in the teaching context, both in Brazil and in Portugal. This fact leads us to propose investment in knowledge of the possible causes of non-adherence to screening.

### Contributions to Nursing

The Health Promoting Universities movement encourages and welcomes multicenter studies, contributing to the adoption of best health self-care practices and community well-being. The sharing of successful experiences, between universities and institutions from different countries, contributes to health interventions enhancing good self-care practices inside and outside the work environment.

## CONCLUSIONS

Moderate and high CVR was associated with high blood pressure, male gender and smoking among participants. The findings corroborate other studies carried out showing that not using tobacco and adhering to a healthy lifestyle predispose to better cardiovascular health. There was no difference in the relationship between lifestyle CVR and CMD, however, the results of screening workers show advantages for early detection of risk and definition of intervention focuses in occupational health to promote health and well-being. Carrying out multicenter studies based on CVR, Lifestyle and CMD screening between universities and institutions from different countries contributed to early identification of community intervention foci and sharing good health care practices, enhancing self-care inside and outside the environment labor.
