# Supplementary material for: Cardiovascular Risk and Lifestyle: comparison between teaching workers in Portugal and Brazil
Source: Rev Bras Enferm. 2024 Jun 14;77(2):e20230354. doi: 10.1590/0034-7167-2023-0354 (PMC11178320; doi:10.1590/0034-7167-2023-0354)
Supplement: Translation

# Translation: Risco Cardiovascular e Estilo de Vida: comparação entre trabalhadores do ensino de Portugal e Brasil

**DOI:** 10.1590/0034-7167-2023-0354pt

**Authors:** Noeli das Neves Toledo, Gilsirene Scantelbury de Almeida, Nair Chase da Silva, Luana Coimbra, Sara Alves Monteiro, Anna Camily Oliveira Bitar, Filipa de Brito Homem, Irma Brito

**Affiliations:** I Universidade Federal do Amazonas. Manaus, Amazonas, Brasil; II Centro Hospitalar e Universitário de Coimbra EPE, Unidade de Cuidados Intensivos Cardíacos. Coimbra, Portugal; III Escola Superior de Enfermagem de Coimbra & UICISA. Coimbra, Portugal

**Keywords:** Risco Cardiovascular, Estilo de Vida, Universidades, Saúde Ocupacional, Pesquisa Participativa Baseada na Comunidade

## Abstract

**Objetivo:**

Comparar o Risco Cardiovascular entre trabalhadores do Brasil e Portugal que atuam no contexto do ensino e sua relação com Estilo de Vida e Transtorno Mental Comum.

**Métodos:**

Estudo transversal que comparou as condições de saúde cardiovascular de trabalhadores do ensino de Manaus (Brasil) e Coimbra (Portugal). Foi estimada a razão de chance entre grupos.

**Resultados:**

As diferenças foram: Tabagismo e hipercolesterolemia nos participantes de Portugal. Hipertensão, doença crônica, perímetro abdominal aumentado, transtorno mental comum e afastamento do trabalho no Brasil. As variáveis com maior efeito para risco cardiovascular alto foram: País-Portugal [17.273 (IC_95%_1.538-193.951)], sexo-masculino [61.577 (IC_95%_5.398 -702.469)] e tabagismo [593.398(IC_95%_57.330- 6.142.020)].

**Conclusão:**

As diferenças de risco entre grupos mostraram que os participantes de Portugal, homens, com pressão arterial aumentada e/ou tabagistas são os mais vulneráveis a ter evento cardiovascular. Há necessidades de intervenções para a promoção da saúde cardiovascular no ambiente laboral nos dois países.

## INTRODUÇÃO

Dados revelam que as Doenças Cardiovasculares (DCV) estão entre as principais causas de mortalidade, sendo 31% de todas as causas de mortes no mundo. Segundo estimativas da OMS, 85% das mortes tiveram como causa principal os enfartes agudos do miocárdio (EAM) e os acidentes vasculares cerebrais (AVC). Embora as prevalências sejam mais elevadas nos países de baixa e média renda, chama a atenção que, dentre os 17 milhões das mortes por doenças crônicas não transmissíveis no mundo, 37% pertencem ao conjunto das DCV e acometeram pessoas com menos de 70 anos de idade^(1,2)^.

Embora gênero, idade, hereditariedade e algumas doenças crônicas (Hipertensão, Diabetes, Dislipidemia e Obesidade) sejam consideradas fatores de risco cardiovascular (FRCV), as condições socioeconômicas (renda/rendimento, nível de escolaridade, profissão) e de estilo de vida (uso de tabaco, álcool, dieta inadequada e sedentarismo) são potencialmente mais relevantes de serem enfrentadas quando o foco é a promoção da saúde e bem-estar do grupo social^(3)^. Essa perspectiva alinha-se no conceito de fatores de risco modificáveis.

Em Portugal e no Brasil estimativas mostraram que a população apresenta elevada prevalência de fatores de RCV contextuais, principalmente no que se refere à adoção de hábitos inadequados e baixo controle dos valores de tensão arterial, glicemia e colesterol^(3,4)^. Como os eventos cardiovasculares têm maior incidência a partir da meia idade, ou seja, em plena idade ativa e produtiva. Essa problemática encaixa-se no âmbito da saúde do trabalhador. Contudo, apesar de existirem programas de saúde ocupacional com exames obrigatórios, os aspectos da saúde e bem-estar são pouco explorados ou são negligenciados, especialmente no que se refere à avaliação de RCV e dos indicadores de bem-estar físico ou mental.

Embora Portugal e Brasil tenham identidades socioculturais próprias, considera-se que as necessidades de cuidados de saúde são semelhantes, principalmente no que se refere à proteção dos fatores de RCV^(3,5,6)^.

Em relação à saúde dos trabalhadores, aspectos da saúde e bem-estar são pouco conhecidos ou estão sendo negligenciados, especialmente no que se refere à prevenção das doenças incapacitantes do ponto de vista físico e mental. Há evidências que esse cenário sofreu piora em detrimento da pandemia pela COVID-19^(7,8)^. Estudos realizados em diversas localidades do mundo mostraram que a população sofreu com o adoecimento e complicações decorrentes do contágio pelo coronavírus, bem como com as mudanças no seu cotidiano social, especialmente no que diz respeito à forma de organização dos processos de trabalho e de acesso aos serviços de saúde^(9,10,11,12)^. Os trabalhadores escolares estão entre as categorias profissionais mais impactadas com tais transformações, sobretudo ao considerar que viven-ciaram um acelerado processo de adaptação tanto no trabalho à distância, no período de confinamento, quanto na retomada das atividades de ensino/aprendizagem para que, assim, as escolas pudessem funcionar de forma satisfatória do ponto de vista quantitativo^(13)^. É importante destacar que a necessidade de reestruturação do processo de ensino-aprendizagem, das formas de atenção psicopedagógica e social – demandadas pelos estudantes que também sofreram com as novas condições de vida impostas pela pandemia – comprometeu as condições de saúde e bem-estar desses trabalhadores nos últimos anos pós pandemia. Nessa perspectiva, este estudo configura-se em melhor conhecer as condições da saúde cardiovascular dos trabalhadores escolares, atuando em duas localidades distintas: Manaus, no estado do Amazonas (Brasil) e Coimbra (Portugal), levando em conta que em ambos os países não há evidências de estudos sobre fatores de risco associados entre os trabalhadores escolares.

Ao considerar que os contextos salutogênicos configuram-se em espaços que geram mais saúde e previnem o adoecimento, este estudo alinha-se à proposta de Pesquisa-ação Participativa em Saúde consubstanciada no modelo PEER--IESS (Participation, Engagement, Empowerment and Research for Innovation and Expansion of Salutogenic Settings) de Instituições de Ensino Superior salutogénicas^(14,15)^. Portugal e Brasil estabeleceram parceria de estudo multicêntrico em contexto escolar. A parceria pretende ativar estratégias dialógicas e criativas de mobilização do público alvo^(14,16,17)^, para ações de pesquisa que permitam traçar o diagnóstico situacional do estilo de vida e bem-estar dos trabalhadores e a co-criação de estratégias salutogênicas. Acordou-se, neste âmbito, realizar um estudo transversal a partir do rastreio de Risco Cardiovascular (RCV), verificando possíveis relações com o Estilo de Vida e presença de Transtorno Mental Comum.

## OBJETIVO

Comparar o Risco Cardiovascular entre trabalhadores do Brasil e Portugal que atuam no contexto do ensino e sua relação com o Estilo de Vida e Transtorno Mental Comum.

## MÉTODOS

### Aspectos Éticos

O estudo foi conduzido de acordo com as diretrizes de ética nacionais e internacionais, sendo aprovado pelos Comitês de Ética da Universidade Federal do Amazonas (UFAM), Brasil, e da Escola Superior de Enfermagem de Coimbra (ESEnfC), Portugal. O Consentimento Livre e Esclarecido foi obtido de todos os indivíduos envolvidos no estudo por meio online.

### Desenho, período e local do estudo

Trata-se de um estudo epidemiológico, observacional que utilizou as recomendações do checklist Strengthening the Reporting of Observational Studies in Epidemiology (STROBE), adaptadas ao tipo de estudo^(18)^.

Parte-se de um projeto multicêntrico PEER-IESS (Participation, Engagement, Empowerment and Research for Innovation and Expansion of Salutogenic Settings), envolvendo Instituições de Ensino do Brasil e de Portugal^(14,15,19,20)^. Descreve-se os resultados de um rastreio de Risco Cardiovascular e sua relação com o Estilo de Vida e Transtorno Mental Comum entre trabalhadores que atuavam no contexto do ensino. A coleta dos dados foi realizada entre agosto de 2022 a maio de 2023 em duas localidades distintas (Manaus – Brasil, e Coimbra – Portugal).

### População, critérios de inclusão e exclusão

O estudo foi realizado com trabalhadores que atuavam no contexto do ensino (docentes e não docentes), em dois países distintos (Brasil e Portugal, conforme já mencionado), que foram adotados como o universo da população-alvo que atendia aos critérios de inclusão e exclusão. Por isso, todos os trabalhadores dos respectivos países e instituições envolvidas foram convidados a realizar a avaliação da sua condição de saúde. As gestantes e os demais trabalhadores que estavam em condição de afastamento por quaisquer que fossem os motivos (férias, licenças: maternidade/paternidade, capacitação ou doença) foram excluídos. O cálculo da amostra estimada considera a frequência antecipada de 50%, o limite de confiança de 5%. O efeito do desenho para estudos com amostras aleatórias de 1.0 foi de 297 para os trabalhadores do Brasil e 197 para Portugal.

Com a finalidade de envolver o público-alvo nas atividades de rastreio, os convites foram enviados para o e-mail institucional dos trabalhadores, no mínimo 2 vezes por semana, durante o período de coleta dos dados (setembro/2022 a junho/2023). Em paralelo, também foram adotadas outras estratégias de mobilização (vídeo de curta duração, webinar, folheto digital e cartazes). Os recursos foram disponibilizados nos sites e redes de comunicação interna de cada instituição envolvida. Apesar de todos os esforços e estratégias para atingir o tamanho amostral, o quantitativo não foi atingido.

Ao considerar o universo de trabalhadores de cada país, a amostra foi constituída de forma aleatória e não probabilística, de modo que todos os trabalhadores pudessem escolher entre participar ou não do estudo, totalizando 203 participantes, sendo 94 do Brasil e 110 de Portugal.

### Protocolo do estudo

A coleta de dados foi realizada por profissionais de saúde e estudantes de enfermagem, todos previamente treinados numa sessão de capacitação.

O primeiro momento da coleta dos dados consistiu em responder ao instrumento que continha perguntas sobre as condições sociodemográficas, laborais, histórico de saúde individual e familiar, bem como estilo de vida (EV) e rastreio de Transtorno Mental Comum - TMC. Para a avaliação do estilo de vida, utilizou-se o questionário denominado: Estilo de vida FANTÁSTICO composto por 25 questões, subdivididas em nove domínios, que são: 1) família e amigos; 2) atividade física; 3) nutrição; 4) cigarro e drogas; 5) álcool; 6) sono, cinto de segurança, estresse e sexo seguro; 7) tipo de comportamento; 8) introspeção e 9) trabalho. As alternativas são na forma de escala Likert ou dicotômicas, sendo a alternativa da esquerda sempre a de menor valor, representando menor associação com um estilo de vida saudável. O somatório dos pontos permite chegar a um escore total que categoriza os indivíduos em cinco grupos: excelente (85 a 100 pontos), muito bom (70 a 84 pontos), bom (55 a 69 pontos), regular (35 a 54 pontos) e necessita melhorar (0 a 34 pontos). Quanto menor o escore, maior será a imprescindibilidade de modificação do estilo de vida^(21)^. A avaliação do TMC foi realizada por meio do Self Reporting Questionnaire (SQR-20). Na versão adaptada, os 20 primeiros itens têm a finalidade de investigar morbidades não psicóticas, como: fadiga, insônia, esquecimento, irritabilidade, dificuldade de concentração, queixas somáticas, humor depressivo/ansioso, decréscimo de energia vital e pensamentos depressivos. A pontuação pode variar de 0 (nenhuma probabilidade) a 20 (extrema probabilidade) de ter TMC, com ponto de corte 5 para o sexo feminino e 7 para o sexo masculino^(22,23,24,25)^.

O segundo momento da coleta dos dados consistiu na avaliação dos seguintes biossinais: glicemia, colesterol, pressão arterial, peso, altura e perímetro abdominal.

Para as medidas capilares da glicemia e do colesterol total, foi utilizado aparelho portátil, com amostra de sangue obtida a partir de uma punção na polpa do dedo indicador do participante. O dispositivo de punção (lanceta) foi de uso individual e descartável. Embora os valores dos exames não configurem diagnóstico médico, foram adotados os seguintes parâmetros de referência:

Glicemia Normal (>126 mg/dl) para os participantes que informaram jejum ≥8 horas. Para os que informaram não estar de jejum foi considerada a glicemia aumentada a partir de valores >200 mg/dl^(26)^. Considerou-se colesterol aumentado quando o participante apresentou valores ≥ 190 mg/dl, independente do tempo de jejum. Em todas as condições de valores alterados o participante foi orientado a repetir o teste^(27)^.

A medida da pressão arterial (PA) foi realizada com aparelho digital de braço. Os participantes considerados com pressão elevada foram aqueles que apresentaram valores da pressão arterial sistólica (PAS) ≥ 140 mmHg e/ou pressão arterial diastólica (PAD) ≥ 90 mmHg durante a medida casual^(28)^.

Os participantes que referiram ter diagnóstico de hipertensão arterial (HA), foram considerados no cálculo da prevalência, ainda que a medida casual da sua PA estivesse dentro dos valores de normalidade.

A medida da estatura, foi realizada através de estadiômetro portátil e o peso em balança portátil, com capacidade máxima de 150kg. Os participantes foram orientados a subir descalços na balança, sendo estimado o peso da roupa (1 quilo) para diminuir o seu peso corporal. O IMC foi classificado em normal (valores entre 18,5 e 24,9kg/m2) ou aumentado (valores ≥ 25 kg/m2)^(29)^.

A medida da circunferência da cintura foi realizada com fita métrica de plástico não extensível (tamanho de 1,5 m). A medida foi feita na circunferência do tronco, na linha do umbigo. O Risco Metabólico para os homens foi classificado em: Risco baixo (PA < 94 cm), Risco aumentado (PA ≥ 94 cm). A mesma classificação para as Mulheres com pontos de corte um pouco menores: Risco baixo (PA < 80 cm), Risco aumentado (PA≥ 80 cm)^(29)^.

Após a avaliação dos biossinais, calculou-se o risco cardiovascular (RCV). A Sociedade Europeia de Cardiologia recomenda que deve ser estimado o RCV global, utilizando o SCORE (Systematic Coronary Risk Evaluation), nas pessoas aparentemente saudáveis com mais de 40 anos. O SCORE é a probabilidade de eventos cardiovasculares fatais em 10 anos e depende da idade, gênero, HA, dislipidemia e tabagismo. Portugal integra o conjunto de países com estimativa de baixo risco, tendo sido adotada a seguinte classificação de risco: *Baixo (< 1%); Moderado (≥1% e <5%); Elevado (≥5% e <10%); Muito elevado (≥10%)* ^(30)^.

Apesar de no Brasil as normas serem ligeiramente diferentes, tendo em vista a comparação dos resultados entre Portugal e Brasil, optou-se por adotar os valores da Sociedade Europeia de Cardiologia para todos os participantes, de ambos os países. Os participantes receberam informações verbais e por escrito dos valores de cada uma das medidas realizadas e o aconselhamento personalizado quanto ao seu risco cardiovascular.

### Análise dos resultados e estatística

Dos dados recolhidos em tabelas de Excel que depois de categorizadas foram transferidas para SPSS, foi realizada a análise estatística descritiva simples, calculando a diferença entre grupos através do teste do Qui-quadrado de Pearson, considerando o nível de significância de 5% e valor de *p* ≤ 0,05. Os efeitos das variáveis Risco Cardiovascular, Estilo de Vida e Transtorno Mental Comum foram estimadas por razão de chance, obtida por regressão logística multinomial, com variância bivariada estimado pelo *Odds Ratio (OR).* Realizou-se, também, o teste de colinearidade que foi ausente para todas as variáveis do modelo. As razões de chance foram apresentadas com o intervalo de confiança de 95% e o valor de p ≤ 0,05. Todos os dados foram analisados com o auxílio de um profissional estatístico

## RESULTADOS

Na comparação das condições sociodemográficas entre trabalhadores do Brasil e de Portugal, é possível observar diferenças quanto à média de filhos, à prática de fé e à renda familiar. Cabe destacar as diferenças dos valores das moedas Real (Brasil) e Euro (Portugal) (R$1,00 para 5,5 €) e respectivos Salários-Mínimos (SM). Assim, o percentual de trabalhadores do Brasil que informaram renda ≥ 8 SM (58,1%) equivale aproximadamente ao percentual dos de Portugal que informou renda entre 1-3 SM (80%) (Tabela 1).

**Tabela 1:** Condições sociodemográficas dos trabalhadores de Portugal e do Brasil, segundo as variáveis: sexo, estado marital, filhos, prática de fé, renda familiar e dependentes da renda. Portugal – Coimbra e Manaus-Brasil, 2022-2023

Variáveis	País		Total 203(100)	Valor de p
	Portugal 110(54,2)	Brasil 93(45,8)		
Sexo ^n(%)^				0,116
Masculino	48 (43,6)	32 (34,4)	80 (39,4)	
Feminino	62 (56,4)	61 (65,6)	123 (60,6)	
Idade(Md/Max-Min)	42 (22-65)	44 (24-67)		0,142
Estado Marital ^n(%)^				0,108
Sem companheiro	28 (25,5)	32 (34,4)	60 (29,6)	
Com companheiro	82 (74,5)	61 (65,6)	143 (70,4)	
Filhos ^n(%)^				0,551
Sim	72 (65,5)	61 (65,6)	133 (65,5)	
Não	38 (34,5)	32 (34,4)	70 (34,5)	
Quantidade de Filhos ^(Md/Max-Min)^	1 (0-4)	2 (0-4)	-	0,019
Praticante de uma fé ^n(%)^				
Sim	55 (50)	70 (24,7)	125 (61,6)	**<0,001**
Não	55 (50)	23 (75,3)	78 (38,4)	
Renda Familiar ^n(%)^				
1-3 Salários-Mínimos	88 (80)	9 (9,7)	97 (47,8)	**<0,001**
4-7 Salários-Mínimos	20 (18,2)	30 (32,3)	50 (24,6)	
≥ 8 Salários-Mínimos	2 (1,8)	54 (58,1)	56 (27,6)	
Dependentes da Renda (Md/Max-Min)	2 (1-6)	2 (1-6)	-	0,873

Em relação às condições laborais, a maioria dos participantes do Brasil era docente (54,8%), enquanto, em Portugal, a maioria era de profissionais técnicos que atuavam no contexto do ensino (66,4%). O tempo de vínculo na instituição foi maior no grupo do Brasil [10 (1-43) anos]. A necessidade de afastamento das atividades laborais foi mais frequente entre os participantes do Brasil (31,2%), sendo a maioria por motivo de doença (82,8%), conforme apresentado na Tabela 2.

**Tabela 2:** Características laborais dos participantes de Portugal e do Brasil, segundo função laboral e tempo de vínculo na instituição, bem como meio de transporte, necessidade e motivo de afastamento do trabalho no último ano. Portugal – Coimbra e Manaus-Brasil, 2022-2023

Variáveis	País		Total 203(100)	Valor de p
	Portugal 110(100)	Brasil 93(100)		
Atividade Laboral^n(%)^				0,002
Docente	37 (33,6)	51 (54,8)	88 (43,3)	
Não docente	73 (66,4)	42 (45,2)	115 (56,7)	
Tempo na Instituição ^(Md/Max-Min)^	7 (1-44)	10 (1-43)	-	0,042
Meio de Transporte^n(%)^				0,481
Carro/moto	98 (89,1)	84 (90,3)	182 (89,7)	
Coletivo	8 (7,3)	8 (8,6)	16 (7,9)	
Caminhando	4 (3,6)	1 (1,1)	5 (2,5)	
Afastamento do Trabalho^n(%)^				0,01
Sim	18 (16,4)	29 (31,2)	47 (23,2)	
Não	92 (83,6)	64 (68,8)	156 (76,8)	
Motivo do Afastamento^n(%)^				0,003
Doença	7 (38,9)	24 (82,8)	31 (66)	
Outros	11 (61,1)	5 (17,2)	16 (34)	

Em relação ao perfil de saúde, pode-se verificar, na Tabela 3, que os dados de colesterol aumentado, consumo de tabaco e RCV moderado e elevado indicaram taxas maiores no grupo de Portugal. Enquanto a HA autorreferida, o histórico de doença crônica e o maior risco de doença metabólica foi no grupo do Brasil. O grupo do Brasil foi o que mais informou ter tido piora na prática de atividade física e comportamento sexual, bem como melhora e piora na alimentação. Além disso, 83,9% dos participantes do Brasil não perceberam melhora em nenhum dos seus hábitos nos últimos 2 anos. O grupo de Portugal teve percentuais mais elevados dos hábitos que melhoram (atividade física e qualidade do sono). Embora os grupos tenham obtido pontuação no EVF consideradas como “Muito Bom” (entre 70 e 84 pontos), a maior média foi nos trabalhadores de Portugal (74,39±9 pontos). Chama a atenção que quase metade dos participantes do Brasil (44,1%) foi classificada como tendo um estilo de vida *Regular* ou a *Melhorar*. Ao considerar as diferenças por sexo, a mediana de pontos de avaliação de TMC foi ligeiramente mais elevada no grupo do Brasil. Contudo, a maior prevalência de presença de TMC foi entre os participantes do Brasil (38,7%).

**Tabela 3:** Perfil de saúde dos trabalhadores de Portugal e do Brasil, segundo autoavaliação de saúde, hábitos e medidas (metabólica, da pressão arterial e antropometria), bem com avaliação do estilo de vida e rastreios de transtorno mental e do Risco Cardiovascular. Portugal – Coimbra e Manaus-Brasil, 2022-2023

Variáveis	País		Total 203(100)	Valor de p
	Portugal 110(100)	Brasil 93(100)		
Autoavaliação da condição de Saúde ^n(%)^				0,031
A melhorar/Regular	71 (64,5)	47 (50,5)	118 (58,1)	
Muito boa/Excelente	39 (35,5)	46 (49,5)	85 (41,9)	
Doença Pregressa ^n(%)^				0,014
Sim	29 (26,4)	39 (41,9)	68 (33,5)	
Não	81 (73,6)	54 (58,1)	135 (66,5)	
Tipos de Doença ^n(%)^				0,277
Doença Cardiovascular	10 (34,5)	9 (47,4)	19 (39,6)	
Outras Doenças Crônicas	19 (65,5)	10 (52,6)	29 (60,4)	
Consumo de Tabaco ^n(%)^				**<0,001**
Sim	31 (28,2)	6 (6,5)	37 (18,2)	
Não	79 (71,8)	87 (95,5)	166 (81,8)	
Medida Casual da Glicemia ^n(%)^				0,563
Normal	108 (98,2)	92 (98,9)	200 (98,5)	
Aumentada	2 (1,8)	1 (1,1)	3 (1,5)	
Diabetes Autorreferida ^n(%)^				0,063
Sim	3 (2,7)	8 (8,6)	192 (94,6)	
Não	107 (97,3)	85 (91,4)	11 (5,4)	
Prevalência de Diabetes ^n(%)^	5 (4,5)	8 (8,6)	13 (6,4)	0,187
Medida Casual do Colesterol ^n(%)^				0,008
Normal	71 (64,5)	75 (80,6)	146 (71,9)	
Aumentado	39 (35,5)	18 (19,4)	57 (28,1)	
Medida Casual da Pressão Arterial ^n(%)^				0,471
Normal	80 (72,7)	69(74,2)	149(73,4)	
Aumentada	30 (27,3)	24(25,8)	54(26,6)	
Hipertensão autorreferida ^n(%)^				0,005
Sim	7 (6,4)	18 (19,4)	25 (12,3)	
Não	103 (93,6)	75 (80,6)	178 (87,7)	
Prevalência de Hipertensão ^n(%)^	31 (28,2)	35 (37,6)	66 (32,5)	0,1
Risco de Doença Metabólica ^n(%)^				0,017
Não	74 (67,3)	48 (51,6)	122 (60,1)	
Sim	36 (32,7)	48,4 (45)	81 (39,9)	
Risco cardiovascular ^n(%)^				0,002
Baixo	59 (53,6)	65 (69,9)	124 (61,1)	
Moderado	36 (32,7)	27 (29)	63 (31)	
Elevado/Muito Elevado	15 (13,6)	1 (1,1)	16 (7,9)	
Hábitos que melhoram ^n(%)^				
Alimentação	42 (38,2)	48 (51,6)	90 (44,3)	0,038
Atividade Física	46 (41,8)	6 (6,5)	52 (25,6)	**<0,001**
Hábitos de sono	21 (19,1)	6 (6,5)	27 (13,3)	0,006
Hábitos Sexual	5 (4,5)	6 (6,5)	11 (5,4)	0,385
Consumo de droga/álcool	6 (5,5)	12 (12,9)	18 (8,9)	0,053
Nenhum	24 (21,8)	78 (83,9)	102 (50,2)	**<0,001**
Hábitos que pioraram^n(%)^				
Alimentação	6 (5,5)	45 (48,4)	51 (25,1)	**<0,001**
Atividade Física	18 (16,4)	45 (48,4)	63 (31)	**<0,001**
Hábitos de sono	42 (38,2)	45 (48,4)	87 (42,9)	0,093
Hábitos Sexual	1 (0,9)	6 (6,5)	7 (3,4)	0,037
Nenhum	50 (45,5)	45 (48,4)	95 (46,8)	0,391
Pontuação do Estilo de Vida – ^FANTÁSTICOM(DV)^	74,39 (9)	69,60 (9,2)	-	**<0,001**
Classificação do Estilo de Vida ^FANTÁSTICO n(%)^				0,005
Excelente a bom ^(>69 pontos)^	82 (74,5)	52 (55,9)	134 (66)	
Regular à Melhorar ^(≤69 pontos)^	28 (25,5)	41 (44,1)	69 (34)	
Pontuação Transtorno Mental Comum^Md (Max-Min)^	3 (0-15)	4 (0-17)	-	0,049
Simn(%)	28 (25,5)	36 (38,7)	139 (68,5)	0,031
Nãon(%)	82 (74,5)	57 (61,3)	64 (31,5)	

As variáveis que tiveram associação simples significativa com a variável RCV foram (Tabela 4): país [risco alto (p < 0,007)], sexo [risco moderado e elevado (p < 0,001)], renda [risco moderado (p < 0,017) e elevado (p < 0,021)], dependentes da renda [risco elevado (p < 0,010)], IMC [risco moderado (p <0,027) e elevado (p < 0,042)], risco de doença metabólica [risco moderado (p < 0,001) e alto (p < 0,014)], HAS [risco moderado (p <0,001], PAS [risco moderado e elevado (p < 0,001)], PAD [risco moderado (p < 0,001) e elevado (p < 0,012)], Tabaco [risco moderado e elevado (p < 0,001)], Piora do sono [risco elevado (p < 0,016)], Estilo de Vida Fantástico [risco moderado (p < 0,009)] e Colesterol [risco moderado (p < 0,016) e elevado (p < 0,001)].

**Tabela 4:** Análise bruta dos fatores associados ao Risco Cardiovascular (Baixo, Moderado, Alto) dos trabalhadores de Portugal e do Brasil. Portugal – Coimbra e Manaus-Brasil, 2022-2023

Variáveis	^#^OR-^##^RCV Moderado vs B aixo ^#^OR ajustada ^[IC95%]^	Valor de p	^#^OR ^##^RCV Alto vs Baixo^#^OR ajustada ^[IC95%]^	Valor de p
País(Portugal)	1.46 (0,797-2.706)	<0,217	16.525 (2.117-128.972)	<0,007
Sexo(Masculino)	9.968 (4.937-20.126)	<0,000	69.545 (20.723-554.459)	<0,000
Renda ^(SM)^				
1-3	2.560 (1.181-5.5450)	0,017	11.408 (1.433-90.840)	0,021
RendaDependentes	0,768 (0,593-0,996)	0,046	0,488 (0,282-0,844)	0,010
IMC*	1.075 (1.008-1.147)	<0,027	1.107 (1.004-1.221)	<0,042
PA**	1.047 (1.020-1.074)	<0,001	1.051 (1.010-1.093)	<0,014
HA(Não)***	0,189 (0,097- 0,368)	<0,000	556 (0,177-1.745)	<0,314
PAS^▴^	1.066 (1.041-1.092)	<0,000	1.067 (1.030-1.105)	<0,000
PAD^▴▴^	1.063 (1.028-1.098)	<0,000	1.067 (1.014-1.122)	<0,012
Tabaco ^(Não)^	0,053 (0,015-0,188)	<0,000	0,004 (0,001-0,023)	<0,000
Pior Sono	6.135 (0,610-0,114)	<0,689	233 (0,071-0,763)	<0,0016
EVF^+^	0,957 (0,925-0,989)	0,009	990 (0,935-1.048)	<0,0724
Colesterol	1.12 (1.002-1.022)	0,016	1.041 (1.025-1.057)	<0,0000

^#^Odds Ratio (OR); ^##^Risco Cardiovascular; ^*^índice de Massa Corporal (IMC); ^**^ Perímetro Abdominal (PA); ^***^Hipertensão Arterial (HA);^▴^Pressão Arterial Sistólica (PAS);^▴▴^ Pressão Arterial Diastólica; + Estilo de Vida FANTÁTISCO (EVF).

Na Tabela 5 estão os resultados do modelo final da Regressão Multivariada, sendo possível observar que os fatores associados ao RCV alto e moderado foram os mesmos, ocorrendo diferenças substanciais nos valores de efeito (*Odds Ratio* – OR). Os participantes de Portugal apresentaram, respectivamente, razão de chance de risco cardiovascular alto e moderado de 17.273 (IC_95%_1.538-193.951] e 1.882 (IC_95%_0,772-4.589). Os participantes do sexo masculino apresentaram razão de chance mais elevada, com 61.577 (IC_95%_5.398-702.469)] para risco alto, e 9.458 (IC_95%_3.877-23.077) para risco moderado. Os que referiram ser tabagistas apresentaram razão de chance extremamente elevada para risco cardiovascular alto 593.398 (IC_95%_57.330-6.142.020)] e moderado [50.594 (IC_95%_9.430-271.462).

**Tabela 5:** Análise ajustada, por Regressão Multivariada, dos fatores associados ao Risco Cardiovascular (Baixo, Moderado e Alto) dos trabalhadores de Portugal e do Brasil. Portugal – Coimbra e Manaus-Brasil, 2022-2023

Variáveis	^#^OR ^##^RCV Moderado vs Baixo ^#^OR ajustada ^[IC95%]^	Valor de p	^#^OR ^##^RCV Alto vs Baixo ^#^OR ajustada ^[IC95%]^	Valor de p
País(Portugal)	1.882 (0,772-4.589)	<0,164	17.273 (1.538-193.951)	<0,021
Sexo(Masculino)	9.458 (3.877-23.077)	<0,000	61.577 (5.398-702.469)	<0,001
PAS^▴^	1.086 (1.051-1.122)	<0,000	1.126 (1.060-1.196)	<0,000
Tabaco ^(Não)^	50.594 (9.430-271.462)	<0,000	593.398(57.330-6142.020)	<0,000

*^#^Odds Ratio (OR); ^##^Risco Cardiovascular ^▴^Pressão Arterial Sistólica (PAS).*

## DISCUSSÃO

Na comparação entre os grupos, os achados mostraram que os participantes do Brasil, tinham prevalências mais elevadas de HA autorreferida (19,4%), histórico de doença crônica (41,9%) e risco metabólico aumentado (45%). No Brasil, as prevalências quanto aos fatores de RCV variam conforme a região do país, sendo possível encontrar prevalências bem mais elevadas de HA (39,3%) e Dislipidemia (64,25%)^(4,31,32)^. Especificamente sobre a prevalência de HA, uma pesquisa por inquérito telefônico, realizada nas principais capitais brasileiras, identificou que ao longo de 2017, 1 em cada 4 adultos evoluiu para o óbito em decorrência das complicações causadas pela doença^(4)^.

Ao comparar os dados nacionais de ambos os países com outros fatores que predispõem o desenvolvimento de HA e outras DCV, verifica-se que as frequências de pré-obesidade (57,2%), obesidade (22,4%), alimentação inadequada (18,2%) e DM (9,1%) são proporcionalmente menores em relação aos percentuais de Portugal [alimentação considerada inadequada (71,3%), pré-obesidade/obesidade (62,1%)], exceto a DM cujo percentual é ligeiramente menor (8,9%)^(3,4)^.

Outros achados importantes dos participantes de Portugal, são as prevalências de colesterol aumentado (35,5%) e consumo de tabaco (28,2%), que estavam mais elevadas em comparação com os participantes do Brasil (19,4%/ 6,5%) e com dados nacionais de Portugal (31,5% e 25,5% respectivamente). Por outro lado, a prevalência de participantes de Portugal que referiram ter diagnóstico de HA (19,4%) foi bem menor que os dados nacionais do Brasil (39,3%) e de Portugal (43,1%)^(3)^.

Considerando a degradação dos estilos de vida para os últimos 2 anos nos dois grupos investigados, podemos inferir que ela pode estar relacionada ao período pandêmico, evidenciando a necessidade de se implementar medidas salutogênicas, ou seja, que o ambiente laboral desenvolva estratégias de incentivo pessoal e coletivo que levem os trabalhadores a adotar boas práticas de autocuidado e, consequentemente, alcançar melhores potenciais de saúde. Urge apoiar as pessoas a restabelecer hábitos saudáveis e evitar o desenvolvimento de doenças crônicas, como: obesidade, HA e DM^(11,12)^.

Quanto ao risco de evento cardiovascular nos próximos 10 anos^(30)^, identificamos que os participantes de Portugal tinham percentuais maiores de RCV moderado (32,7%) e elevado (13,6%), quando comparados aos do Brasil. As variáveis associadas ao aumento da razão de chance de risco cardiovascular alto e moderado foram entre os participantes de Portugal, do sexo masculino e, principalmente, aqueles que referiram consumir tabaco.

Um estudo epidemiológico, realizado em Portugal, concluiu que os 5 fatores de RCV, são: pré-obesidade/obesidade, HA, dislipidemia, nível baixo de atividade física e tabagismo, sendo que mais da metade população (68%) apresenta no mínimo ≥ 2 fatores de RCV, no qual a HA e o DM são considerados doenças de maior dificuldade de controle^(3)^. Na realidade brasileira, as estimativas mostraram que o RCV aumentou com a idade e na população de baixa renda. A hipertensão, hipercolesterolemia, diabetes e tabagismo, são fatores de RCV em ambos os sexos^(33)^.

Os dados deste estudo revelam dois importantes focos de atenção para intervenções de promoção da saúde e bem-estar: a falta de adesão a um estilo de vida saudável, especialmente no que se refere ao uso de tabaco. Os participantes de ambos os países apresentaram condição de vulnerabilidade no que se refere à saúde cardiovascular. Embora os fatores de risco sejam amplamente descritos na literatura científica, os rastreios para avaliação do RCV são vistos como estratégias potencializadoras na prevenção e controle dessas doenças, pois otimizam a implementação de tratamento precoce, principalmente nas pessoas que possuem risco mais elevado^(31,32,33,34,35,35)^. Ao considerar que o consumo de tabaco é um problema não só do grupo de participantes de Portugal, mas da maior parcela da população de ambas as nações, inclusive entre os mais jovens, entende-se que implementar e promover intervenção de cessação tabágica é uma das estratégias que permite reduzir drasticamente a probabilidade de DCV^(36)^.

O estudo que avaliou a saúde cardiovascular (CV) e sua relação com o estresse de trabalhadores universitários de Rio Branco -Brasil identificou que nenhum dos participantes atendeu aos critérios da saúde CV ideal (dieta, atividade física, IMC, tabagismo, hipertensão, diabetes e hipercolesterolemia). A maioria (91%) foi classificada com saúde CV baixa e o estresse no trabalho foi associado à obesidade [OR 2,11 (IC95% 1,06-4,22; p = 0,034)] e à dieta inadequada: [OR 2,31 (IC95%: 1,29-4,13; p = 0,005)]^(37)^.

Além disso, uma outra pesquisa internacional, que comparou a saúde CV da população de 5 países de alta à baixa renda (Inglaterra, EUA, Brasil e Etiópia), identificou que o Brasil obteve a menor pontuação na saúde CV (7,7/12), sendo que apenas 38,7% da população foi considerada com saúde CV ideal. Em contraste, a Etiópia obteve a maior pontuação (10,5/12), visto que 91,2% da população estava com saúde CV ideal. Na comparação dos achados do Brasil com os países de alta renda (Inglaterra e EUA), as metas ideais de valores do IMC (42,5%), colesterol total (63,7%) e tabagismo (84,5%) foram alcançadas com maior prevalência pelo Brasil. Por outro lado, os EUA superam o Brasil quanto ao percentual de pessoas com valores normais da PA (43,5%) e nível adequado de atividade física (59,8%). A Inglaterra obteve o melhor desempenho entre as três nações nas variáveis: glicemia (75,5%), atividade física (70%) e PA (66,3%)^(38)^.

Nesse contexto, destaca-se que o movimento das Universidades Promotoras de Saúde (UPS), ao permitir o fortalecimento de uma cultura organizacional de cuidado, envolvendo a comunidade universitária, pode potenciar múltiplas e recorrentes ações que contribuam na adesão a um melhor estilo de vida. Segundo o modelo PEER-IESS^(19,39)^, as instituições de ensino também podem agregar valor ao princípio de indissociabilidade entre ensino, pesquisa e extensão, quando desenvolvem ou promovem intervenções pautadas na abordagem da Pesquisa-Ação-Participativa em Saúde (PaPS). Esta abordagem vai além da recolha de dados científicos porque propõe a adoção de estratégias dialógicas e criativas que potencializam a capacidade da comunidade universitária de enfrentar os seus problemas, aumentar a sua literacia em saúde e tornar os ambientes mais salutogênicos^(15,^40^)^.

### Limitações do Estudo

Foi considerado como limitação do estudo a diferente adesão dos trabalhadores: docentes de Portugal e técnicos administrativos do Brasil. Embora a estimativa do tamanho amostral não tenha sido atingida, os achados na amostra são preocupantes, sendo possível generalizar que os fatores de RCV são semelhantes entre os trabalhadores que atuam no contexto do ensino, tanto no Brasil como em Portugal. Esse fato leva-nos a propor investimento no conhecimento das possíveis causas de não adesão ao rastreio.

### Contribuições para a Enfermagem

O movimento das Universidades Promotoras de Saúde incentiva e acolhe os estudos multicêntricos, contribuindo para a adesão de melhores práticas de autocuidado de saúde e bem-estar da comunidade. O compartilhamento de experiências exitosas, entre universidades e instituições de países distintos, contribui para que as intervenções de saúde potencializem boas práticas de autocuidado dentro e fora do ambiente laboral.

## CONCLUSÕES

ORCV moderado e alto associou-se à hipertensão arterial, ao sexo masculino e ao tabagismo entre os participantes. Os achados corroboram com outros estudos realizados evidenciando que o não uso de tabaco e a adesão a um estilo de vida saudável predispõem a melhor saúde cardiovascular. Não houve diferença na relação entre RCV estilo de vida e TMC, entretanto, os resultados do rastreio dos trabalhadores evidenciam vantagens para detecção precoce de risco e definição de focos de intervenção em saúde ocupacional para promoção da saúde e bem-estar. Realizar os estudos multicêntricos que tenham por base o rastreio RCV, Estilo de vida e TMC entre universidades e instituições de países distintos contribuiu para identificar precocemente os focos de intervenção comunitária e partilhar boas práticas de cuidado em saúde, potencializando o autocuidado dentro e fora do ambiente laboral.
